# Experimental and numerical study of steady state stability in a toluene biodegrading biofilter

**DOI:** 10.1038/s41598-022-15620-w

**Published:** 2022-07-22

**Authors:** Michael Süß, Alex De Visscher

**Affiliations:** 1 Department of Chemical and Petroleum Engineering, and Centre for Environmental Engineering Research and Education (CEERE), Schulich School of Engineering, University of Calgary, 2500 University Drive NW, Calgary, AB T2N 1N4 Canada; 2 Department of Chemical and Materials Engineering, Gina Cody School of Engineering and Computer Science, Concordia University, 1455 De Maisonneuve Blvd. W, Montreal, QC Canada

**Keywords:** Chemical engineering, Pollution remediation

## Abstract

Different steady states in a toluene biodegrading biofilter were explored experimentally and numerically. Experimental results showed that a gradual increase of the toluene inlet concentration over several weeks leads to a consistently low exit concentration, with a drastic increase at an inlet concentration change from 7.7 to 8.5 g m^−3^, indicating an alteration in steady state. A significant and sudden drop in the removal efficiency from 88 to 46% was observed. A model that includes nitrogen and biomass dynamics predicted results matching the experimental biofilter performance well, but the timing of the concentration jump was not reproduced exactly. A model that assumes a gradual increase of toluene inlet concentration of 0.272 g m^−3^ per day, accurately reproduced the experimental relationship between inlet and outlet concentration. Although there was variation between experimental and simulated results, a clear confirmation of the jump from one steady state to another was found.

## Introduction

Gaseous emissions from various industrial processes need to be treated before they are released into the environment because of their harmful effect on humans, animals, and the environment. Waste gas streams potentially contain hazardous substances such as volatile organic compounds (VOCs) or odorous compounds. The oil and gas, chemical and pharmaceutical industries are anthropogenic sources of such VOCs. The need to comply with stringent legislation can pose a challenge, because any waste gas treatment system must be reliable, sustainable, and efficient. Physical and chemical methods are applied in industry but are often associated with disadvantages such as operational costs as well as the generation of undesirable by-products compared to their biological counterpart^1–4^. The treatment of polluted air by means of biological techniques is known and well-established^5–11^. In a biofilter, microbes degrade the compound of interest and produce water, carbon dioxide and heat^2,4^.

A biofilter simulation enables the prediction of the behavior and efficiency of a system and is a substantial part of biological air pollution control research in recent decades. It is an auxiliary method to aid biofilter designers and manufacturers, as they link biological growth kinetics to reaction engineering.

Ottengraf and Van Den Oever^12^ developed one of the first models in 1983. In their study, experimental results corresponded well with a model that assumes mass transfer limitation and zero-order kinetics. Since then, numerical based approaches, sometimes supported by experimental data of biofilter and biofilm kinetics were developed in the last decades^13–20^. These models overcome some of the limitations of the Ottengraf and Van den Oever model. Yan et al.^21^ studied the effect of the filter bed porosity on removal efficiency. They showed a low impact (i.e., high efficiency) at low Darcy numbers (< 10^–4^), whereas at high Darcy numbers, the porosity significantly affects the removal efficiency. Woudberg et al.^22^ proposed a pore-scale model to predict the pressure drop of a biofilter. The prediction of Malakar et al.^23^ returned to the original Ottengraf and Van Den Oever model to describe the theoretical elimination capacity and theoretical average biofilm thickness in a toluene biofilter. The biofilm was assumed to be static in this study. Dorado et al.^24^ estimated the kinetic parameters of a more sophisticated model involving biofilm diffusion and Monod kinetics. Biofilm thickness was also assumed to be static in this study. With continuous digitalization and increasing available biofiltration data, the implementation of machine learning approaches to predict the biofilter performance is now possible^25^.

In Süß and De Visscher^26^, it was shown that a sudden drop in removal efficiency (RE) potentially is an implication of different steady states occurring in the biofilm if (1) the pollutant concentration exceeds a threshold value and (2) substrate degradation and substrate inhibition follow Haldane kinetics. However, the dynamics and implication of steady states in a biofilm or biofilter aerobically treating gaseous contaminants are not yet fully understood, mainly because existing biofilter models do not include the dynamic nature of biofilm growth and die-off during biofiltration. In this study, we hypothesize that biofilters can progress from a single steady state behavior to a multi steady state behavior, driven by the growth of the biofilm thickness as the biofilter is exposed to increasing pollutant concentrations. At the early stare of biofilter operation, biofilms are too thin to display multiple steady states. As biofilms grow thicker, the filter goes through a phase of high activity at conditions where a second, low-activity steady state exists. At this stage, a slight concentration increase can cause the biofilter to “crash”, i.e., to drop from the high activity to the low activity steady state.

To test this hypothesis, a toluene biofiltration experiment was run with inlet concentrations gradually increasing to high levels (> 10 g/m^3^), and a model was developed with a dynamic biofilm thickness driven by nitrogen availability for biomass to grow on toluene.

## Materials and methods

### Microorganism and filter bed material of biofilter

A sterilized mixture of compost and wood chips (vol% 80/20) was used as filter bed. In Table 1, the filter bed analysis is shown. Analysis was conducted by AGAT Laboratories. Chemicals such as toluene and solids for the cultivation medium were obtained from Sigma Aldrich. An adapted liquid BH-medium^17^ was used as a growth medium and contained 1 g L^−1^ KH_2_PO_4_, 1 g L^−1^ Na_2_HPO_4_, 0.5 g L^−1^ NH_4_NO_3_, 0.002 g L^−1^ FeCl_3_, 0.002 g L^−1^ MnSO_4_·2H_2_O, 0.2 g L^−1^ MgSO_4_·7H_2_O and 0.02 g L^−1^ CaCl·2H_2_O. The medium was sterilized before use.

**Table 1:** Filterbed properties.

Filterbed properties			
pH		7.30	–
Particle size Distribution			
	(2000–50 μm)	44	%
	(50–2 μm)	37	%
	(< 2 μm)	19	%
NO_3_-N and NO_2_-N		916	mg kg^−1^
Total nitrogen		15,900	mg kg^−1^
PO_4_-P		194	mg kg^−1^
Organic matter		29.50	%

Toluene was used as a single carbon source. The growth medium was used to culture a toluene degrader, *Nocardia* sp.^17^. To introduce additional microorganisms into the inoculant, an air stream was piped into the serum bottles. After 3 days, plating of cells was conducted, and standard procedures were followed^27^. Two other bacterial strains were found. These other two bacteria were tested on their ability to biodegrade toluene as a single carbon source by firstly isolating and secondly growing them in serum bottles with Toluene as only carbon source. No microbial growth was determined hence, these microbes were not able to biodegrade toluene. Therefore, it is assumed that *Nocardia* sp. is the only Toluene-degrading microbe in the mixture and that the other strains were autotrophic bacteria. No further characterization of microbes was conducted. *Nocardia* sp. as Toluene-degrading and two non-Toluene degrading microbes were cultivated under ambient temperature (21–22 °C) in serum bottles sealed with a butyl rubber septum and aluminum crimp.

### Biofilter set-up and experimental conditions

The Toluene biofiltration experiments were carried out in a lab-scale biofilter with a packing as described above, inoculated with 100 mL of inoculant (OD_650_ around 0.2). The column was made of polycarbonate with a total height of 63 cm and an internal diameter of 10 cm. At the bottom, glass beads at a height of 5 cm and a 1 cm perforated Plexiglas plate were used to evenly distribute the inlet gas stream. Sampling ports to measure gas samples were centered at the top and bottom of the column and sealed with GC septa (0.95 cm diameter). The biofilter was filled with sterilized filter media, containing dispersed inoculant to a height of 25 cm and placed in a fume hood under ambient temperatures of 21–22 °C. The experimental set-up is based on^17^. An air pump (pond master Ap-40) was used to generate one air stream, which was split and sent through two gas washing bottles, one filled with tap water (resistivity 0.0029 MΩ, hardness 169 mg/L as CaCO_3_, pH 7.8), and one filled with the Toluene. Subsequently, the two gas flows were combined and mixed in an empty gas-washing bottle before being introduced into the biofilter. The flow was controlled by two rotameters (Cole-Parmer) located after the gas flow split, and total flow rate was measured (TI-400) before the sample port and inlet.

### Biofilter performance parameters

The removal efficiency (RE), inlet loading rate (ILR), the elimination capacity (EC), and the empty-bed residence time (EBRT) are commonly used to describe the performance of a biofilter and are defined as follows:

1 $${\text{RE}} = \frac{{\text{C}}_{\text{i}} - \text{C}_{\text{o}}}{{\text{C}}_{\text{i}}} * 100$$

2 $${\text{ILR}} = \frac{\text{Q C}_{{\rm i}}}{\text{V}}$$

3 $${\text{EC}} = \frac{{\text{Q}} (\hbox{C}_{{{\rm i}}} -{\text{C}}_{\text{o}})}{\text{V}}$$

4 $${\text{EBRT}} = \frac{\text{V}}{{\text{Q}}}$$

where C_i_, C_0_, Q, and V represent the inlet concentration [g m^−3^], outlet concentration [g m^−3^], volumetric flow rate [m^3^ h^−1^] and biofilter volume [m^−3^], respectively.

### Biofilter operation

An air stream contaminated with toluene was treated under different operational conditions. An EBRT of 4.5 min was maintained and a stepwise increase of the inlet concentration was conducted. This EBRT is higher than typical but not unusual when the inlet concentration is high^28–30^. An adsorption test for toluene was conducted with sterilized filter packing and indicated no adsorption.

The operational parameters of the biofilter experiments are shown in Table 2.

**Table 2:** Used model parameters for all simulations^33,34^.

Used model parameters	2 steady sates experiment	Steady increase of inlet concentration	Biofilter experiment	Units
Collocation points (biofilm)	10	10	10	–
Henry's constant (H)	0.25	0.25	0.25	–
Initial biomass (X_0_)	0.003	0.003	0.003	g_biomass_ kg_compost_^−1^
Specific surface area of biofilm (A)	0.95	1.1	0.2375	m^2^ kg^−1^
Yield (Y)	0.5	0.5	0.5	g g^−1^
µ_max_	0.1	0.1	0.1	h^−1^
V_max_	0.3	0.3	0.3	g g^−1^ h^−1^
Decay rate (a)	0.0014	0.0014	0.0014	h^−1^
Superficial velocity (υ_s_)	3.63	3.63	3.63	m h^−1^
Organic nitrogen content (N_org_)	14.98	14.98	14.98	g_N_ g_compost_
Half-saturation constant K_m_	0.05	0.05	0.05	g m^−3^
Inhibition constant K_I_	2.7	2.7	2.7	g m^−3^
Nitrogen uptake rate constant (k_uptake_)	0.0022	0.0022	0.0022	h^−1^
Nitrogen mineralization constant (k_min_)	0.00007	0.00007	0.00007	h^−1^
Michaelis–Menten constant for nitrogen (K_N_)	0.5	0.5	0.5	g_N_ kg_compostdw_^−1^
Mass fraction of nitrogen in toluene degrading biomass (f_N_)	0.126	0.126	0.126	g_N_ g_biomass_^−1^
Porosity of the biofilter (ε)	0.55	0.55	0.55	–
Diffusivity in biofilm (D)	1.5E−10	1.5E−11	1.5E−12	m^2^ s^−1^
Density of bulk (ρ_bulk_)	555	555	306	kg_compost_ m_biofilter_^−3^
Density of biofilm (ρ_bio_)	1.00E+05	1.00E+05	1.00E+05	g m^−3^

### Analytical methods

An SGE 250 µL gastight syringe was used to draw and inject 200 µL of gas sample from the gas sampling ports and into the analyzer. The gas samples were analyzed with a gas chromatograph (GC-2014, Shimadzu) equipped with an FID and Rtx®-Wax capillary column (30 m × 0.53 mm × 1 µm). The injector and detector temperatures were set at 250 °C. The oven temperature for toluene was 80 °C. Helium was used as a carrier gas.

### Model description

The multiplicity of steady states in an aerobic biofilter is rarely studied and understood. Simulations with a biofilm model by Süß and De Visscher^26^ showed that different steady states can exist inside a biofilm, considering no other back-mixing mechanisms. It was also established that a change in steady state can lead to a sudden decrease in RE for a small increase of the pollutant concentration. To further investigate the possible steady states in a biofilter, a full biofilter model with biofilm dynamics was developed based on a number of assumptions about the biofilter and the biofilm:

1. Gas phase flow is assumed to behave in plug flow pattern. Thus, axial dispersion is neglected.
2. Due to low gas phase concentration, the gas-biofilm equilibrium at the interface is described by Henry’s law.
3. A planar geometry of the biofilm is assumed.
4. Haldane kinetics is assumed to describe substrate biodegradation and substrate inhibition.
5. Oxygen is not considered as a limiting factor.
6. Inorganic nitrogen cycling is considered in the model and described in^17^.
7. Diffusion of toluene into the biofilm follows Fick’s law.

### Model development

To minimize computation time of the model, orthogonal collocation^31^ was used both in the gas phase and in the biofilm phase, linked together by using Henry’s law at the biofilm surface. In order to predict the outlet concentration of the biofilter, the packing material was divided into 25 collocation points along the biofilter height. For each collocation point in the gas phase, the gas-phase concentration was calculated, and a biofilm consisting of 10 collocation points was modeled to calculate the average reaction rate, net growth rate, and biofilm concentration profile. In addition, a nitrogen cycle was also considered in the model^32^. Matlab was used to solve the model equation. Numerical integrations of ordinary differential equations were conducted with Matlab function ode15s.

Fick´s law is used to describe molecular diffusion in this model:

5 $${\text{J}} = - {\text{D}}_{\text{A}} \, \frac{{\text{dC}}_{{\rm A}}}{\text{dx}}$$

where J, D_A_, C_A_ and x refer to the diffusive flux of component A [g_Substrate_ m^−2^ h^−1^], the diffusion coefficient of component A [m^2^ h^−1^], the concentration of the compound A [g_Substrate_ m^−3^] and the length coordinate [m] in the direction of the biofilm thickness. The reaction rate was calculated with Haldane kinetics, which includes substrate inhibition, as follows:

6 $${\text{r}} = - \frac{{\text{V}}_{\text{max}} \, {\text{C}}_{\text{A}} \, \uprho_{\text{bio}}}{{\text{K}}_{\text{s}} + {\text{C}}_{\text{A}} + \frac{{{\text{C}}_{\text{A}}}^{2}}{{\text{K}}_{\text{I}}}}$$

V_max_ is the activity parameter [g_substrate_ g_dw Substrate degrading biomass_^−1^ h^−1^], K_s_ is the affinity parameter [g_substrate_ m^−3^], K_I_ is the inhibition parameter [g_substrate_ m^−3^] and ρ_bio_ reflects the biomass density of microorganisms in the biofilm [g_dw_ m^−3^].

In order to calculate the concentration profile in the biofilm, diffusion and reaction rate, Equations (5) and (6), were linked together, considering the biofilm thickness L [m], the distance coordinate in the biofilm x [m] and a dimensionless distance coordinate in the biofilm x′ (= x/L) using a material balance, which leads to the following expression.

7 $$\frac{\partial\text{C}_{\text{Abiofilm}}}{\partial\text{t}} = \frac{{\text{D}}_{\text{A}}}{{\text{L}}^{2}} \, \frac{{\partial}^{2}{\text{C}}_{\text{A}}}{\partial{\text{x}^{\prime}}^{2}} - \frac{{\text{V}}_\text{max }{\text{C}}_{\text{A}}}{{\text{K}}_\text{s }+ \text{ C}_{\text{A}} + \frac{{\text{C}}_{\text{A}}{2}}{{\text{K}}_{\text{I}}}}\uprho_{\text{bio}}$$

To solve the above equation, the following boundary conditions were used:

8 $${\left.{\text{C}}_{\text{A}}\right|}_{{\text{x}}^{{\prime}}= 1 }=\frac{{\text{C}}_{\text{Agas}}}{\text{H}}$$

9 $${\left.\frac{{\partial\text{C}}_{\text{A}}}{\partial{\text{x}}^{\prime}}\right|}_{{\text{x}}^{{\prime}}= 0 }= 0$$

where the inside boundary of the biofilm away from the gas is represented by x = 0. The partial differential equation in Eq. (7), which is first-order in time, and second-order in space, was solved by Orthogonal Collocation to approximate the concentration profile in the biofilm and as outlined by Villadsen and Stewart^31^. Next, the concentration in the gas phase in each collocation point was computed by equating the transfer of toluene in the gas phase towards the biofilm to the integrated toluene biodegradation in the biofilm.

10 $$\frac{{\text{dCc}}_{\text{Agas}}}{\text{dt}}= \frac{{-\upupsilon }\frac{{\text{dC}}_{\text{Agas}}}{\text{dz}}- \uprho_{\text{bulk}}\text{ A L }\overline{\text{r}}}{\upvarepsilon}$$

where

11 $$\overline{\text{r}}= \underset{0}{\overset{1}{\int }}\frac{{\text{V}}_{\text{max}}\text{ C}_{\text{Abiofilm}}\uprho_{\text{bio}}}{{\text{K}}_{\text{s}}+ \text{C}_{\text{Abiofilm}}+ \frac{C_{\text{Abiofilm}}^{2}}{{\text{K}}_{\text{i}}}}{\text{dx}}^{\prime}$$

Here υ expresses the superficial velocity [m h^−1^] and A the specific surface area of the biofilm [m^2^ kg^−1^].

To account for the biofilm growth, the net growth rate of the microorganism needs to be considered. As mentioned above, the nitrogen cycle is considered in this model and a part of it is expressed in the following equation:

12 $$\upmu = \left(\frac{\upmu_\text{max }{\text{C}}_{\text{Agas}}}{{\text{K}}_{\text{S}}+ \text{C}_{\text{Agas}}+ \frac{C_{\text{A}}^{2}}{{\text{K}}_{\text{I}}}}\right) \, \left(\frac{{\text{N}}_{\text{inorg}}}{{\text{k}}_\text{N\_Nitrogen}+ \text{N}_{\text{inorg}}}\right)$$

13 $$\upmu_{\text{net}}= \upmu - {\rm a}$$

where, µ_max_, µ_net_, N_inorg_ k_N_Nitrogen_ and a, express the maximum growth rate [h^−1^], the net growth rate [h^−1^], inorganic nitrogen concentration of the packing material [g_N_ kg_compostdw_^−1^], the Michaelis–Menten constant for nitrogen utilization [g_N_ kg_compostdw_^−1^] and the decay rate [h^−1^], respectively. The growth of the biofilm as a function of time is described by

14 $$\frac{\text{dX}}{{\text{dt}}} = \upmu_{\text{net}}\text{ X}$$

where X is the biomass concentration [g_dwbiomass_ kg_compost_^−1^].

The consumption rate of inorganic nitrogen, r_N_, is calculated as follows:

15 $${r}_{N}= \overline{\mu }{\text{f}}_{\text{N}}\,{\rho}_{\text{bio}}\,\text{ A L}$$

where f_N_ is the nitrogen fraction of the microorganisms, and $\overline{\mu }$ is the average growth rate over the biofilm, calculated in a manner similar to $\overline{r }$. A is the specific area of the biofilm [m^2^ kg_compost_^−1^]. The dynamics of inorganic nitrogen is calculated as follows and shown in^32^:

16 $$\frac{{\text{d}}_{\text{Ninorg}}}{\text{dt}}= \text{k}_{\text{minN}}\text{ N}_\text{org }\text{- k}_{\text{uptakeN}}\text{ N}_\text{inorg }- {r}_{N}$$

where N_inorg_, k_minN_ and k_uptakeN_ represent the inorganic nitrogen content in the packing material [g_N_ kg_dw_^−1^], the nitrogen mineralization rate constant [h^−1^], and the nitrogen uptake rate constant [h^−1^], respectively.

The thickness of the biofilm, L, is calculated with the following equation:

17 $$L=\frac{X}{A\cdot {\rho }_{bio}}$$

## Results and discussion

### Experimental data—steady state

**Figure 1:**
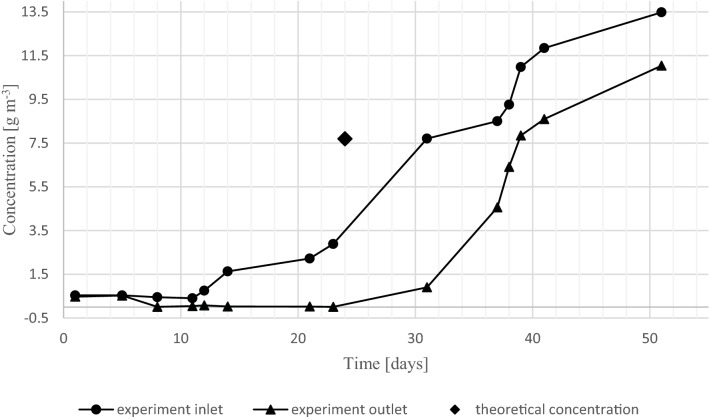
Experimental results of toluene biofiltration in conditions designed to yield two steady states (EBRT = 4.5 min). The single data point depicted as a rhomboid, represents the theoretical increase in the biofilter inlet after increasing the inlet concentration; i.e., it marks the time when the inlet concentration was changed to the level measured on day 31.

In Fig. 1 the inlet and corresponding outlet concentrations of the main experiment, are displayed to evaluate the occurrence of two steady states. The standard deviation of the exit concentration measurements was 0.10 g m^−3^ (i.e., a standard error of 0.056 g m^−3^ based on triplicate measurements). Until day 31, the outlet concentration barely changed, although the inlet concentration was gradually increased. At this time a decline of the RE from 99 to 88% was measured as the inlet concentration was increased by 4.9 g m^−3^ (increase from 2.9 to 7.7 g m^−3^). The inlet concentration was adjusted and increased prior to day 31 to allow the biomass to adjust to the new load prior to the measurement. This is indicated in Fig. 1, 3 and 4 with a theoretical calculated inlet concentration depicted as a rhomboid. The next increase of the inlet concentration was by 0.8 g m^−3^ (to a value of 8.5 g m^−3^), which led to a steep increase of the outlet concentration. This corresponds to a decrease of RE from 88 to 46%. Such a significant decline in RE for a small increase in inlet concentration could indicate that a boundary of a stable steady state region has been crossed.

As discussed and numerically shown in Süß and De Visscher^26^ with a biofilm model, a change in steady state can be explained by substrate degradation and substrate inhibition following Haldane kinetics and the diffusion behavior of the pollutant into the biofilm. Distinctive for Haldane kinetics is that low reaction rates occur at low and high concentrations and high reaction rates occur at medium concentrations. With regard to the conducted experiments, the transition from high to low reaction rates is in the range of 7.7–8.5 g m^−3^. Furthermore, diffusion limitations are an important factor as well. When medium range concentrations are present at the surface of the biofilm, it is possible to maintain such concentrations throughout the biofilm, leading to a high reactivity and pronounced diffusion limitation. Hence, a significant concentration gradient will be upheld in the biofilm and will consequently maintain a medium range concentration at the inside of the biofilm. On the other hand, if a high enough concentration is present at the surface of the biofilm, reaction rates near the surface are low. Thus, a high concentration can develop throughout the biofilm and therefore result in low reactivity. In this case, diffusion limitation is not pronounced.

### Fitting computer simulation to experimental data—steady states

**Figure 2:**
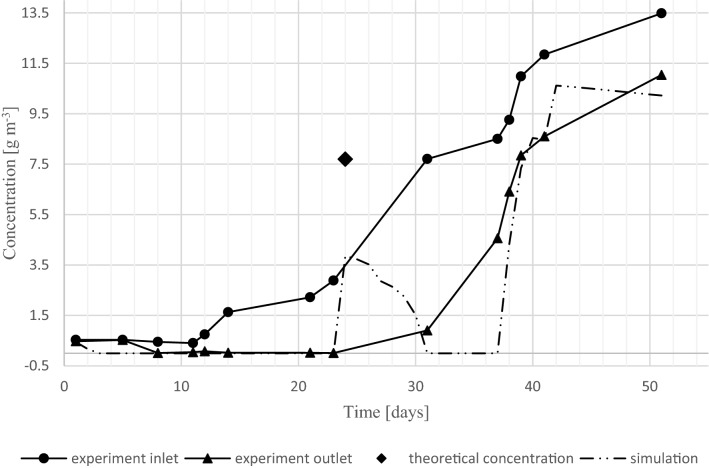
Experimental results and model prediction of toluene biofiltratin (EBRT = 4.5 min). The single data point depicted as a rhomboid, represents the theoretical increase in the biofilter inlet after increasing the inlet concentration—the actual measurement at the inlet was carried out a later day.

The computer simulation developed here was used to predict the outlet concentrations based on obtained inlet concentrations of the experimental trial. To optimize the model, parameters A and k_min_ were used as adjustable variables. The remaining parameter values were taken from previous studies^33,34^. Used model parameters are displayed in Table 2 and the simulation results and experimental data are shown in Fig. 2.

The model prediction and the experimental results are in good agreement until day 23. After that day, the predicted outlet concentration suddenly rose and declined over a period of 8 days. As indicated above, the inlet concentration was increased on day 24, and the exit concentration measurement was made on day 31. This is indicated in Fig. 2, with the expected inlet concentration after adjustment depicted as a rhomboid. That increase of the inlet concentration caused the predicted sudden rise at the outlet. The subsequent predicted decline of outlet concentration is possible due to the adaption of the system to the high stepwise increase of the inlet concentration (biofilm growth). During the next time period (day 31 to 37) the experimental trial indicates a change in steady states, whereas the model prediction indicates such a change between day 37 and 38, at a concentration change from 8.502 to 9.257 g m^−3^. This corresponds to a 47% decline in RE. Between day 37 and 40 the maximum achieved decline in RE is 72%. The root mean square error (RMSE) between the modeled and measured outlet toluene concentration is 1.39 g m^−3^ (r^2^ = 0.900). Although the change in steady state is not predicted in the same time period and concentration range, an indication of change can be seen. A further adjustment of model parameters could possibly increase the accuracy of the model. Alternatively, it may be the case that the nitrogen dynamics model of Eqs. (15–16) does not fully capture the dynamics of biofilm development, particularly under rapidly changing conditions. If this is the case, then it can be expected that a model with a more gradually increasing inlet concentration can describe the relationship between inlet and outlet toluene concentration more satisfactorily.

**Figure 3:**
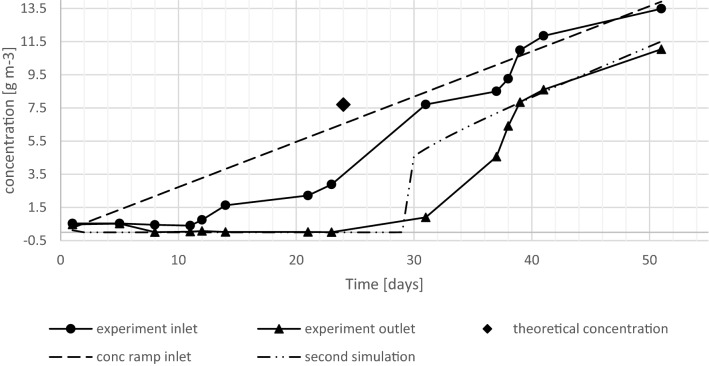
Experimental results and predicted outlet concentration at steady increasing of inlet concentration at steady increase of inlet concentration.

To test this hypothesis, a second model run was carried out, where the inlet concentration was steadily increased with uniform increments of 0.272 g m^−3^ per day. The results are shown in Fig. 3, overlaid with the experimental data. To optimize the model fit, the surface area (A) was increased slightly from 0.95 to 1.1 m^2^ kg^−1^.

As depicted, the predicted change in outlet concentration and the corresponding RE are barely changing until an inlet concentration of 7.909 g m^−3^ is reached on day 29. Then the RE declined from 99.93 to 43.79% on day 30 at a concentration change of 0.272 g m^−3^. Another stepwise increase of inlet concentration led to a further decrease of RE to 40.39%. Henceforth a continuous and slow decrease of RE can be observed based on the increasing outlet concentration. When the modeled sudden decrease of RE (from 99.93% to 43.79%) is compared to the experimentally obtained results, a similar behavior can be observed, with a small inlet concentration change of 0.8 g m^−3^ in the experiment leading to a decrease of the RE from 88 to 46%. This behavior is indicated in Fig. 3 on day 30 for the model and day 37 for the experiment. Consequently, in both cases, simulation and experimental results, a change in steady state occur at similar concentration. The modeled jump is sharper than the observed jump. This is because the model assumes cross-sectionally uniform biofilm thickness, whereas the actual biofilm will not be uniform within a cross-section. The inlet concentration before the jump was 7.909 g m^−3^ for the simulation and 7.7 g m^−3^ for the experiment, and the inlet concentration after the observed jump were 8.181 g m^−3^ and 8.5 g m^−3^ for the simulation and experiment, respectively. When expressed as a function of time, the RMSE between the model and the experimental results is 1.36 g m^−3^ (r^2^ = 0.907).

**Figure 4:**
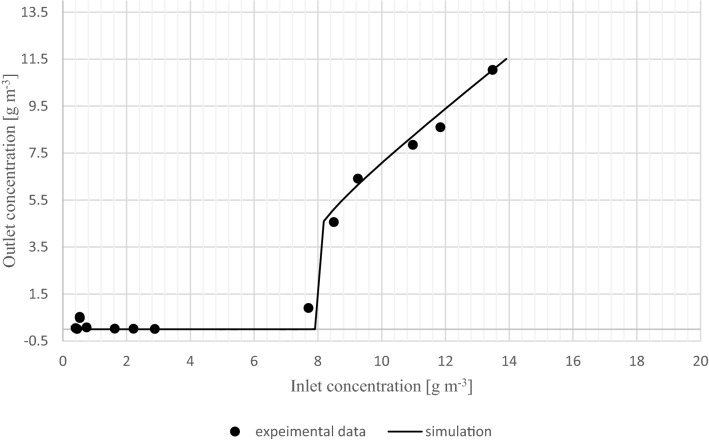
Outlet concentration versus inlet concentration of experimental trial and simulation with steady increase of inlet concentration.

Figure 4 is included to better illustrate the similarity between the experimental data and the simulation shown in Fig. 3. In Fig. 4 the outlet concentration is plotted versus inlet concentration. A very good agreement is obtained (RMSE = 0.40 g m^−3^, r^2^ = 0.992). In this figure, a significant increase in the outlet concentration is observed at a small change in inlet concentration.

This indicates a jump from a high activity steady state to a low activity steady state. The observed behavior should not be confused with a collapse of the biofilm. A collapsing biofilm at such a low inlet concentration change would occur over an extended time (days to weeks), not suddenly, considering no other changing factor or inhibition.

**Figure 5:**
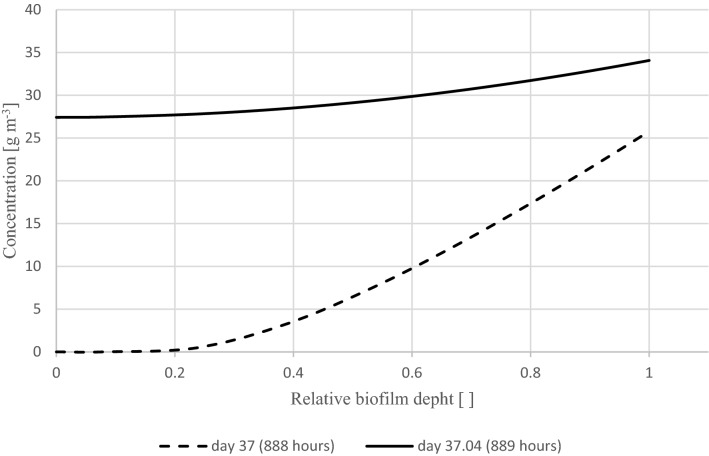
Predicted concentration in the biofilm by using the experimental inlet concentration, showing a sudden change from non-saturated to saturated biofilm within a single hour.

In the above simulation, the concentration in the biofilm is low when high activity is predicted and high when low activity is shown. This indicates a non-saturated and saturated biofilm, respectively. This is illustrated in Fig. 5, where the concentration profile in the biofilm is shown at the beginning of day 37 (888 h) and one hour later (889 h), in biofilter grid point 6 (i.e., at a biofilter height of 3.25 cm), simulating the experimental conditions. The concentration is plotted as a function of distance from the solid surface of the packing material to the surface of the biofilm. As can be seen, the concentration sharply declines towards the inside of the biofilm on day 37, which indicates a non-saturated biofilm and high activity. One hour later, on day 37.04, the concentration in the biofilm has settled to a new steady state associated with a nearly saturated biofilm and lower activity.

### Fitting computer simulation to experimental data—biofilter

**Figure 6:**
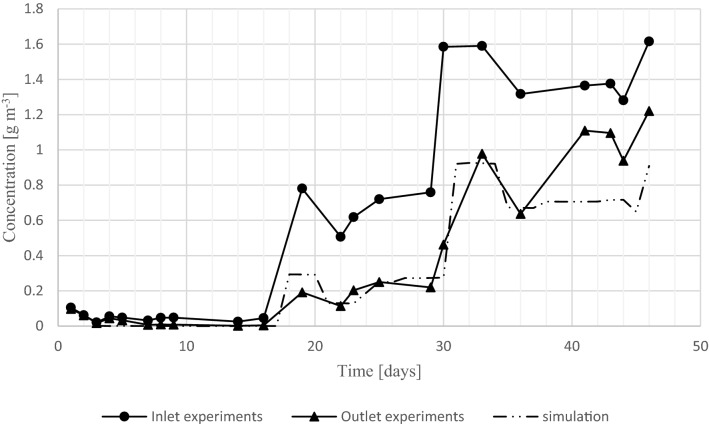
Experimental inlet and outlet concentration and predicted outlet concentration.

To further validate the accuracy and applicability of the computer simulation, it was verified against a second set of biofilter experiments^33^. In this experiment, the inlet concentration was increased stepwise and held at each stage until a steady state was reached. In Fig. 6 the predicted outlet and experimental inlet and outlet concentrations are shown, and the model parameters are listed in Table 2. The experimental outlet concentrations have a standard deviation of 0.071 g m^−3^ (standard error of triplicates 0.041 g m^−3^). To reach a good fit between the model and the data, the specific surface area was reduced by a factor 4. This corresponds with increasing the packing size from about 6 mm to about 25 mm (assuming spherical particles and a solid density of 1000 kg m^−3^). These are reasonable values, and the increase was expected because the straw used as a bulking agent in these experiments did not sustain the structure of the biofilter as well as the wood chips used in the first experiment.

The model follows the experimental data well until day 36. After that, the model underpredicts the outlet concentration. It is hypothesized that settling of the biofilter reduced the biofilm specific surface area exposed to the gas phase. Some slight settling was observed in this biofilter, unlike the biofilter of the data in Fig. 1. The reduced specific surface area reduced the biofilm area in the biofilter. Despite the lack of fit towards the end of the experiment, the model showed a RMSE of only 0.153 g m^−3^ (r^2^ = 0.937). When only the first 36 days are considered, the RMSE is 0.059 g m^−3^ (r^2^ = 0.950). The simulations indicate that the biofilm is strongly diffusion-limited in this biofilter, so a direct proportionality between biofilm area and activity is expected. The assumption of proportionality between activity to specific surface area is consistent with Delhoménie et al.^35^, who found that maximum EC decreased with increasing particle size, but increases with increasing specific surface area. This implies that at that point in time the biofilm surface area has a more pronounced impact on the RE then the activity of the biofilm.

These findings are closely linked to the moderately hydrophobic nature of toluene. Zhu et al. ^36^ found that a toluene biofilter has an degradation efficiency intermediate between isobutanol (H = 0.0005) and n-hexane (H = 53), indicating partial diffusion limitation, consistent with our model interpretation of the results. Nevertheless, biofiltration can be effective at Henry’s law constants of 10 and above (Haque et al.,^37^). Ranjbar and Gheeni ^38^ found that the Henry constant and the specific surface area were the most sensitive parameters in their biofilter model, consistent with our findings. Kalantar et al.^39^ found that toluene is more strongly diffusion-limited in a two-phase biotrickling filter than was found in our work. However, biotrickling filters have thicker biofilms than biofilters, which explains the difference.

The accuracies of the model predictions presented here are similar to the accuracies that are typical for biofilter models, such as Ranjbar and Ghaemi^38^, and San Valeo et al.^40,41^.

## Conclusion

Experimental and simulation results of a toluene biofilter with a steadily increasing inlet concentration show a jump from a high- to low-activity steady state, albeit at a slightly different timing. Results showed a good overall prediction of the outlet concentration, except at the end of the experiment, where settling of the filter bed material may have reduced the biofilm area. An investigation of modeled toluene concentration profile in the biofilm before and after the sudden jump in RE confirmed that the cause of the jump is a transition from a diffusion-limited high-activity state to a low-activity state.

## Data Availability

The datasets used and/or analysed during the current study available from the corresponding author on reasonable request.

## List of symbols

A: Biofilm specific surface area (m_biofilm_^2^ m_biofilter_^−3^)
a: Decay rate of biomass (h^−1^)
C_A_: Substrate concentration (g m^−3^)
Ce: Exit substrate concentration in the gas phase (g m^−3^)
C_i_: Inlet substrate concentration in the gas phase (g m^−3^)
D_A_: Diffusion coefficient of substrate in the biofilm (m^2^ h^−1^)
EBRT: Empty bed residence time (h)
EC: Elimination capacity (g m^−3^ h^−1^)
f_N_: Mass fraction of nitrogen content in toluene degrading biomass (g_N_ g_biomass_^−1^)
H: Dimensionless Henry’s law volatility constant (–)
ILR: Inlet loading rate (g m^−3^ h^−1^)
K__min_N_: Rate constant for nitrogen mineralization (h^−1^)
K__uptake_N_: Rate constant for nitrogen uptake (h^−1^)
K_I_: Kinetic constant for substrate inhibition in the liquid phase (g m^−3^)
K_m_: Michaelis–Menten constant for substrate biodegradation in the liquid phase (g m^−3^)
K_N_: Michaelis–Menten constant for nitrogen (g_N_ kg_compost__dw_^−1^)
N: Number of Collocation Points
N_inorg_: Inorganic nitrogen content in compost (g kg_compost__dw_^−1^)
N_org_: Organic nitrogen content in compost (g kg_compost__dw_^−1^)
Q: Volumetric flow rate of waste gas (m^−3^ h^−1^)
RE: Removal efficiency (%)
V: Biofilter bed volume (m^3^)
V_max_: Maximum reaction rate (g m_biofilm_^−3^ h^−1^)
X: Biomass concentration (g_dw__biomass_ kg_compost__dw_^−1^)
Y: Yield (g g^−1^)
δ: Biofilm thickness (m)
ε: Porosity of the packed bed
μ_max_: Maximum specific growth rate of biomass (h^−1^)
μ_net_: Net specific growth rate of biomass (h^−1^)
ρ_bio_: Density of biofilm (g_dw__biomass_ m_biofilm_^−3^)
ρ_bulk_: Bulk density (kg_compost_ m_biofilter_^−3^)
υ: Superficial gas velocity (m h^−1^)
